# A Robust Homogeneous Fluorescence Polarization Immunoassay for Rapid Determination of Erythromycin in Milk

**DOI:** 10.3390/foods12081581

**Published:** 2023-04-07

**Authors:** Changfei Duan, Huiyan Zhang, Yingjie Zhang, Qiang Li, Peipei Li, Ghulam Mujtaba Mari, Sergei A. Eremin, Jianzhong Shen, Zhanhui Wang

**Affiliations:** 1 National Key Laboratory of Veterinary Public Health Security, Beijing Key Laboratory of Detection Technology for Animal-Derived Food, College of Veterinary Medicine, China Agricultural University, Beijing 100193, China; 2 Department of Veterinary Pharmacology and Toxicology, Faculty of Bio-Sciences, Shaheed Benazir Bhutto University of Veterinary and Animal Sciences, Sakrand 67210, Pakistan; 3 Department of Chemistry, M.V. Lomonosov Moscow State University, 119991 Moscow, Russia

**Keywords:** erythromycin, fluorescence polarization immunoassay, homogeneous detection, milk

## Abstract

Erythromycin (ERY) is one of the most common macrolides applied in veterinary medicine to treat diseases or as a feed additive for animal growth promotion. Long-term irrational use of ERY could lead to residues in animal-derived food and the emergence of drug-resistant strains, posing potential threats to human health. In this study, a highly sensitive, specific, robust, and rapid fluorescence polarization immunoassay (FPIA) for the determination of ERY in milk has been described. Herein, to achieve high sensitivity, five tracers of ERY with different fluorescein structures were synthesized and paired with three monoclonal antibodies (mAbs). Under the optimized conditions, the combination of mAb 5B2 and tracer ERM-FITC achieved the lowest IC_50_ value in the FPIA with 7.39 μg/L for ERM. The established FPIA was used to detect ERY in milk, revealing a limit of detection (LOD) of 14.08 μg/L with recoveries of 96.08–107.77% and coefficients of variations (CVs) of 3.41–10.97%. The total detection time of the developed FPIA was less than 5 min from the addition of samples to the result readout. All the above results showed that the proposed FPIA in this study was a rapid, accurate, and simple method for the screening of ERY in milk samples.

## 1. Introduction

Erythromycin (ERY) was the first macrolide class antibacterial introduced in 1952, which is produced by *Streptomyces erythrues* and possesses antimicrobial activity [1]. ERY is one of the most common macrolides applied in veterinary medicine to treat respiratory diseases and enteric infections in swine, cattle, and poultry, and is employed to treat clinical or subclinical mastitis in lactating cows [2]. It has been used as a feed additive or in drinking water in large-scale production for animal growth promotion [3]. The long-term irrational use or non-compliance with the withdrawal period of ERY could lead to the presence of its residues in the animal-derived food and pose a potential threat to human health through provoking allergic reactions or causing antibiotic-resistant bacteria. Bacterial antibiotic resistance is a serious public health issue and infections caused by antibiotic-resistant bacteria can be difficult and sometimes impossible to treat. ERY-resistant bacteria also have been extensively disclosed and growing rates of ERY resistance have been observed in many different countries [4,5,6,7]. In addition, the risk of human intoxication still existed, although ERY is considered largely free of serious toxicity. The metabolism of ERY is related to the cytochrome P-450 3A isozymes, and inhibition of P-450 3A by medications may elevate the concentrations of ERY in plasma, thereby increasing the risk of ventricular arrhythmias and sudden death [8]. Based on these risks of drug resistance and intoxication, it is necessary to monitor the residues of erythromycin in animal-derived foods.

The maximum residue limit (MRL) for ERY in milk has been set at 40 μg/kg by China and the European Union [9,10] while the Food and Drug Administration (FDA) has set the MRL for ERY in milk at 0 μg/kg [11]. Thus, there is a high pressure on laboratories responsible for food safety to monitor the use of these drugs. Various instrumental methods have been established for the determination of ERY in food samples [1,12,13,14,15,16]. These instrumental methods for monitoring ERY are generally time-consuming, requiring skilled technicians and complex sample pretreatment, which may not always be available for high-throughput screening. Thus, sensitive, specific, robust, and rapid screening methods are urgently needed for effective monitoring of ERY in food.

**Figure 1:**
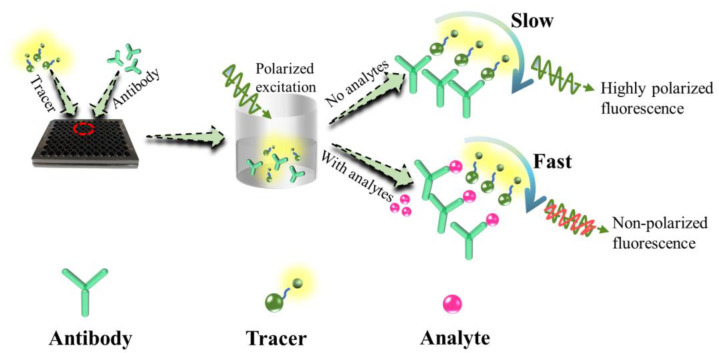
Schematic representation of the FPIA for detecting analytes.

Immunoassay techniques are increasingly applied for screening harmful contaminants in food samples due to its sensitivity, selectivity, and time efficiency, and have become an effective alternative to instrumental methods. However, compared with heterogeneous immunoassay, i.e., Enzyme-Linked Immunosorbent Assay (ELISA), homogeneous immunoassays for operation simplification has shown great potential for routine applications [17]. Fluorescence polarization immunoassays (FPIAs) are one of the most extensively used homogeneous techniques with the advantage of sensitivity, reliability, rapidity, and suitability for the analysis of a large numbers of samples [18,19]. The principle of FPIA for small molecules is that the tracer (fluorescein-labeled competing antigen), competitor (analyte), and antibody react with each other, resulting in a change of the fluorescence polarization (FP) value as shown in Figure 1 [20]. If there are no analytes in the reaction solution, the binding of the tracer and specific antibody forms a bulky antigen–antibody complex with slower movement, resulting in a higher FP value. With an increase in the concentration of analytes in the reaction solution, the antibody will be occupied by analytes, and the tracer will be bound to little or no antibody and some tracers will be free, which can lead to the decrease of the FP value. FPIAs have attracted more attention and been reported for the detection of many small molecular compounds, such as veterinary drugs [18,21,22,23], pesticides [24], and toxins [25,26,27] in food and environmental samples. However, to the best of our knowledge, no report of FPIA for ERY is available.

In this work, five tracers were acquired by conjugation of ERY and ERM haptens with five fluoresceins. We investigated the effects of mAb–tracer pair, mAb–tracer concentration, physicochemical factors, and reaction time on the performance of the FPIA. After careful optimization, a highly robust and rapid FPIA for the detection of ERY was established. The accuracy and precision of the FPIA was then investigated by detecting ERY in milk.

## 2. Materials and Methods

### 2.1. Reagents and Apparatus

Erythromycin (ERY), spiramycin (SPI), josamycin (JOS), dirithromycin (DIR), clarithromycin (CLA), and oleandomycin (OLE) were supplied by TCI Chemicals Ltd. (Shanghai, China). Roxithromycin (ROX), erythromycin ethyl succinate (ERE), ivermectin (IVM), and avermectin (AVE) were acquired from Dr. Ehrenstorfer Gmbh (Ausburg, Germany). Erythromycylamine (ERM), tylosin (TYL), and kitasamycin (KIT) were purchased from GLPBIO (Montclair, CA, USA). Valnemulin (VAL) and tiamulin (TAM) were obtained from the Council of Europe’s European Pharmacopoeia (Strasbourg, France). 4′-aminomethyl fluorescein (4′-AMF) was supplied by Life Technologies., Ltd. (Eugene, OR, USA). Fluorescein isothiocyanate (FITC) and dichlorotriazine aminofluorescein (DTAF) were purchased from Sigma-Aldrich (St. Louis, MO, USA). Sulforhodamine 101 sulfonyl chloride (SRSC) was supplied by Bridgen Biotechnology Ltd. (Beijing, China). Alexa Fluor 647 Succinimidyl Ester (AF647) was supplied by Thermo Fisher Scientific Inc. (Waltham, MA, USA). N′-hydroxysuccinimide (NHS), 1-ethyl-3-(3-dimethylaminopropy) carbodiimide (EDC), O-carboxymethyl oxime (CMO), and N,N-dimethylformamide (DMF) were supplied by Aladdin Chemistry Co., Ltd. (Shanghai, China). The other reagents supplied by Sinopharm Chemical Reagent Co., Ltd. (Shanghai, China).

Precoated TLC silica gel GF254 glass plates (100 × 100 mm) were supplied by Qingdao Haiyang Chemical Branch (Shandong, China). The opaque microplates (96-well) were supplied by Corning Life Science (Oneonta, NY, USA). The microplate reader SpectraMax M5 (Sunnyvale, CA, USA) was used to record the fluorescein intensity (FI) and fluorescence polarization (FP). MAbs 5B2, 6C2, and 6D9 against ERY were produced by our laboratory [28].

Borate buffer (BB, 0.05 M, pH 8.0) was employed as the diluent buffer. A stock standard solution (2 mg/mL) was dissolved in 1 mL methanol.

### 2.2. Synthesis and Characterization of Tracers

#### 2.2.1. Synthesis and Purification of Tracers

**Figure 2:**
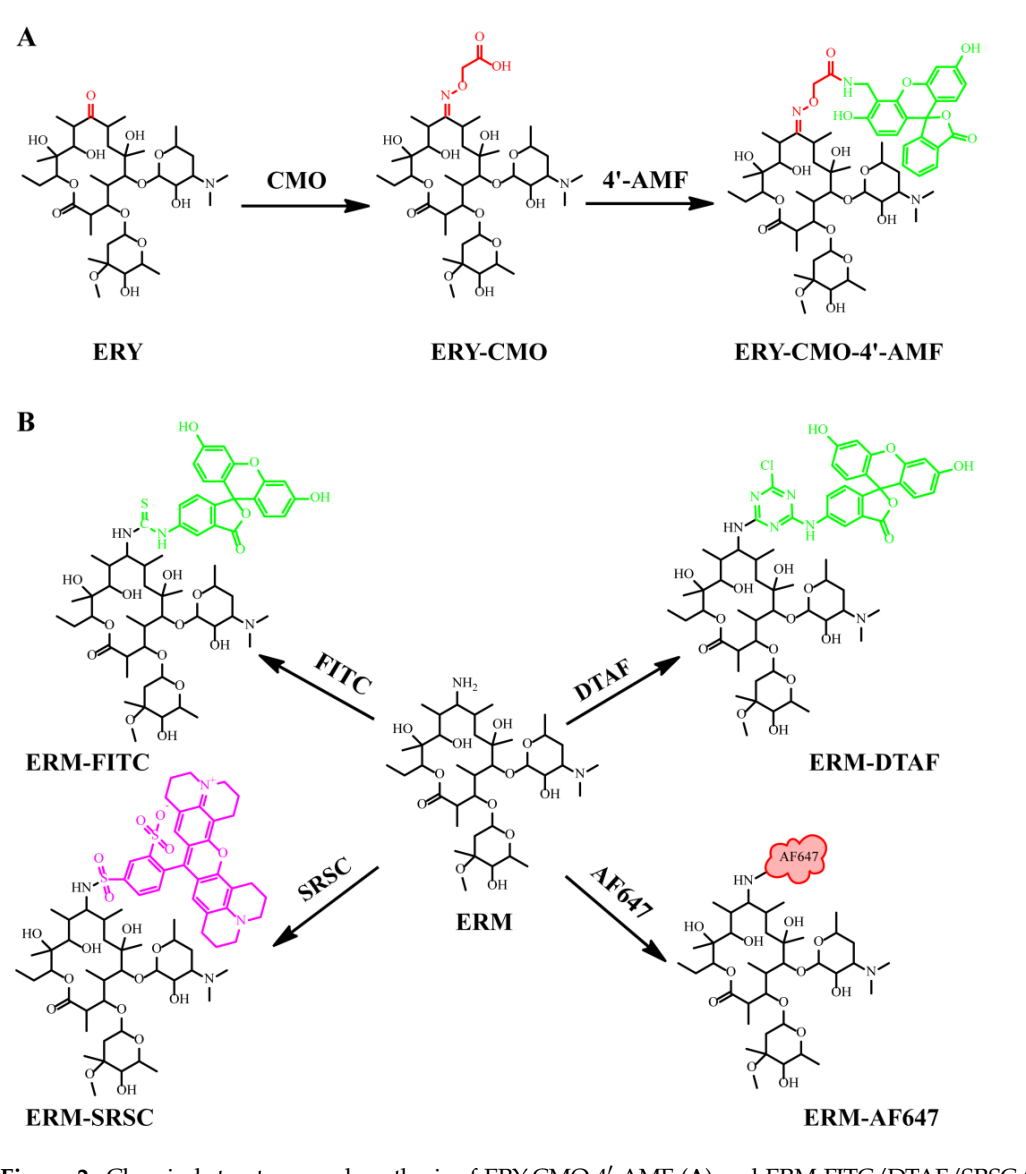
Chemical structures and synthesis of ERY-CMO-4′-AMF (**A**) and ERM-FITC/DTAF/SRSC/AF647 (**B**).

ERY-CMO was previously prepared [28] and conjugated to amino-fluorescein (4′-AMF) using the active ester method (Figure 2A). Briefly, NHS (2 mg) and EDC (4 mg) were prepared with 1 mL DMF. ERY-CMO (200 μL) and 200 μL of DMF were pooled and react at room temperature (RT) for 8 h with stirring. Thereafter, 4′-AMF (2 mg) and triethylamine (50 µL) were added into the activated ERY-CMO tube, and stirred at RT in dark conditions overnight. The crude product (50 μL) and fluorescein control product (10 μL) were purified by TLC using dichloromethane/methanol (1:3, *v*/*v*) as the developing solvent. The major yellow band that was different from the fluorescein control product was collected with methanol and then stored at 4 °C.

ERM-FITC was synthesized according to our previously published method with minor modifications (Figure 2B) [29]. Briefly, ERM (1 mg) was solubilized in 1 mL DMF, and then FITC (1 mg) and triethylamine (50 µL) were added to 200 μL of the above solution and react at RT for seven days in dark conditions. The upper liquid was collected and separated by TLC, and then developed with dichloromethane/methanol (1:1, *v*/*v*). The major yellow band obtained for ERM-FITC was scraped from the TLC plate and extracted using methanol. ERM-DTAF, ERM-SRSC, and ERM-AF647 were separated following the same methods, and the major bands were obtained.

#### 2.2.2. Characterization of Tracers

Tracers were confirmed by HPLC-MS/MS and the saturated antibody binding experiment. For the saturated antibody binding experiment, all the obtained tracers were first diluted with BB to acquire a working solution (FI values were approximately 50) and the FP values for the free tracers were measured (denoted as mP_min_). An aliquot of 70 µL of tracers, 70 µL of 1/100 diluted antibody (mAb 5B2 as representative), and 70 µL of BB were pooled at RT, followed by the measurement of FP values for the bound tracers (denoted as mP_max_). The binding between antibodies and tracers was evaluated by measuring the maximum polarization shift (δmP = mP_max_ − mP_min_). The FP values were recorded for ERY-CMO-4′-AMF, ERM-FITC, and ERM-DTAF at λ_ex_ 485 nm and λ_em_ 530 nm (cutoff 515 nm); ERM-SRSC at λ_ex_ 540 nm and λ_em_ 580 nm (cutoff 570 nm); and ERM-AF647 at λ_ex_ 644 nm and λ_em_ 685 nm (cutoff 665 nm). The binding of mAb 5B2 and the five tracers was measured, and the FP of each tracer that observably changed were utilized for further study.

### 2.3. Development of FPIA for ERM

#### 2.3.1. The mAb Dilution Curve

Three mAbs against ERY were double diluted from 1/100 to 1/51,200 in BB and then mixed with a working tracer solution. Briefly, 70 µL of tracer with 70 μL of mAb and 70 µL of BB were pooled in a well, and then incubated at RT to measure the change in FP. Then, the antibody dilutions curves and δmP were plotted and measured. For the developed FPIA, the antibody titer was the maximal dilution value to produce 50% tracer binding.

#### 2.3.2. The Calibration Curves

The competitive calibration curves for the FPIA were carried out by detecting ERY as follows: 70 µL of the working tracer, 70 µL of serially diluted ERY standard solution, and 70 µL of the working mAb against ERY was pooled After mixing at RT, the FP values of the reaction mixture were recorded. Finally, according to our previous study, competitive calibration curves were generated by plotting the FP values against the logarithm of the ERY concentrations and fitted to a four-parameter logistic equation [30]. The IC_50_ is considered as the concentration of ERY which inhibited 50% of tracer binding to its corresponding mAb. In addition, IC_20_–IC_80_ was defined as the assay’s dynamic range.

#### 2.3.3. The FPIA Optimization

To improve the sensitivity of the assay, the effects of the concentrations of the tracer and mAb, pH, and reaction time were investigated by evaluating the ratio of IC_50_ and δmP (IC_50_/δmP) obtained from the competitive calibration curves. The parameters showed that the lower IC_50_/δmP was desirable.

#### 2.3.4. Specificity Evaluation of the FPIA

The specificity of the FPIA was assessed and analyzed under the optimized conditions. Cross-reactivity (CR) was studied with the following equation:

CR = (IC_50_ of ERY) ⁄ (IC_50_ of tested macrolides) × 100%

where the IC_50_ values were measured from the competitive calibration curves for ERY and each of the tested macrolides.

The limit of detection (LOD) of the FPIA was described as the standard concentration that the average value of 20 independent blank controls plus three times their standard deviation (mean + 3SD).

#### 2.3.5. Preparation of the Milk Samples

The milk samples were spiked with ERY at concentrations of 50, 100, and 150 μg/L, then an equal volume of 10% acetonitrile–BB was added. After being vortexed for 5 min, the samples were allowed to stand at RT for 30 min. The mixtures were centrifuged for 10 min at 10,000× *g*. The supernatant was collected and diluted six-fold with BB and then the concentrations of ERY in the milk samples was measured by FPIA.

## 3. Results and Discussions

### 3.1. Synthesis and Characterization of Tracers

In this study, ERY-CMO was obtained using ERY modified by CMO at the C9 position and introducing a reactive carbonyl group as previously described [28]. Tracer synthesis is important for the establishment of a highly sensitive and specific FPIA, once the antibody is prepared [29,31]. Therefore, to achieve highly sensitive detection of ERY, five new tracers including ERY-CMO-4′-AMF, ERM-FITC, ERM-DTAF, ERM-SRSC, and ERM-AF647 were designed using two haptens and five fluoresceins (Figure 2). After the separation and purification by TLC, the primary bands for ERY-CMO-4′-AMF with a R_f_ of 0.7, ERM-FITC with a R_f_ of 0.5, ERM-DTAF with a R_f_ of 0.56, ERM-SRSC with a R_f_ of 0.66, and ERM-AF647 with a R_f_ of 0.58 were collected (Figure S1). Due to the unclear structure of fluorescein, ERM-AF647 was not subjected to mass spectrometry, but the other four tracers were validated with mass spectrometry. The mass spectrometry results of three tracers were successfully obtained and the molecular ion peaks (*m*/*z*) were 1151.7 for ERY-CMO-4′-AMF, 1125.7 for ERM-FITC, and 1324.7 for ERM-SRSC in positive ion mode (Figure S2A–C); the identification of the tracer ERM-DTAF failed.

**Figure 3:**
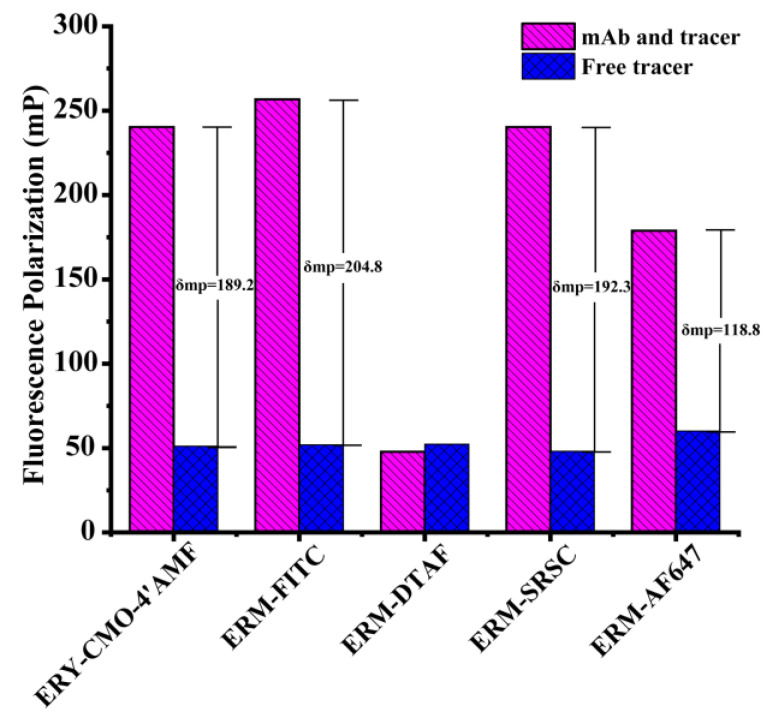
Results of the tracers ERY-CMO-4′-AMF, ERM-FITC, ERM-DTAF, ERM-SRSC, and ERM-AF647 binding with mAb 5B2.

Five tracers were further determined by the FPIA. As shown in Figure 3, ERY-CMO-4′-AMF, ERM-FITC, ERM-SRSC, and ERM-AF647 showed obvious binding to the 1/100 diluted mAb 5B2 with an δmP of 189.2, 204.8, 192.3, and 118.8, respectively. The tracer ERM-DTAF showed no significant change in FP values before and after the binding to the mAb 5B2. The above data suggested that the synthesis of the four tracers (ERY-CMO-4′-AMF, ERM-FITC, ERM-SRSC, and ERM-AF647) could be used to develop the FPIA.

### 3.2. Selection of the mAb–Tracer Pairs

The antibody and tracer pairs are the most crucial elements in the sensitivity of the FPIA. Antibody titers reflect the binding affinity between the antibody and the tracer. Three mAbs (5B2, 6C2, and 6D9) were paired with all tracers and investigated. The antibody titers of the three mAbs with four tracers were acquired by the antibody dilution curves (Table 1 and Figure S3A–D). Tracers ERY-CMO-4′-AMF, ERM-FITC, and ERM-AF647 supplied enough increase in the detection signal for 109–225 mP and high antibody titers, indicating a stronger recognition between the antibody and the tracer. In addition, the tracer ERM-AF647 showed a higher antibody dilution (from 1/3000 to 1/10,000) than others, followed by ERY-CMO-4′-AMF, and ERM-FITC.

The competitive calibration curves of the FPIA for ERY were established by screening the most sensitive mAb–tracer pairs (Figure S4A–D). The IC_50_ of all the calibration curves of the mAb–tracer pairs are shown in Table 1. The tracers ERM-AF647 and ERM-FITC paired with mAb 5B2 showed the higher sensitivities with IC_50_ values of 13.0 ng/mL and 24.0 ng/mL, respectively. The result clearly suggested that the optimized combination of mAb and tracer successfully improved the sensitivity of the FPIA. For instance, the IC_50_ from the pair of mAb 6C2 and ERM-SRSC (IC_50_ of 390.0 ng/mL) were 30-fold higher than that from the pair of mAb 5B2 and ERM-AF647. The IC_50_ from the pairs of mAb 5B2 with ERM-FITC and ERM-AF647 were similar, but after considering the cost of fluorescein (e.g., 20 CNY/mg for FITC, and 5900 CNY/mg for AF647 from Thermo Fisher Scientific Inc., Waltham, MA, USA), the pair of mAb 5B2 and ERM-FITC was employed as the mAb–tracer pair and was utilized in the next experiment.

### 3.3. Development and Optimization of the FPIA for ERY

In FPIA, limited tracer and antibody concentrations are considered to achieve the desired performance [29]. In addition, the dyes applied in this study were pH-sensitive reagents, and the combination between the mAb and tracers could be significantly affected by buffer pH [31,32]. Thus, the influence of these factors was assessed by contrasting the IC_50_/δmP ratios achieved under different experimental conditions.

#### 3.3.1. Optimization of the Tracer Concentration

**Figure 4:**
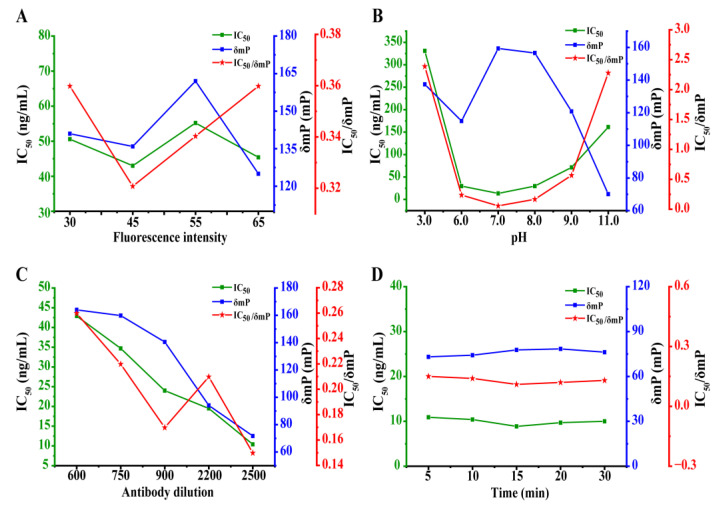
The optimization of the FPIA for ERY. (**A**) Optimization of the tracer FI; (**B**) effect of pH; (**C**) optimization of the antibody dilution; (**D**) optimization of the reaction time.

The tracer concentration was empirically utilized at the beginning of this work, which defined as the concentration when its FI value was about 10-fold higher than the FI value of BB. The FI value of the BB used was approximately 2.8. Therefore, the FI values of the tracer concentrations at 30, 45, 55, and 65 were assessed. As can be seen in Figure 4A, the lowest IC_50_/δmP was screened when the FI of ERM-FITC was 45.

#### 3.3.2. The Effect of the Buffer pH

The combination of the antibody and tracer could be significantly affected by pH. To evaluate the influence of pH, BB was adjusted to different pH values. As observed in Figure 4B, detrimental effects on the IC_50_ and δmP were shown when the pH was higher or lower. However, no obvious effect was demonstrated from pHs ranging from 6.0 to 8.0. The lowest IC_50_/δmP of 0.08 was acquired at pH 7.0.

#### 3.3.3. Optimization of the mAb 5B2 Concentration

Different concentrations of mAb at 1/600, 1/750, 1/900, 1/2200, and 1/2500 were supplied to pursue the best IC_50_/δmP. The results depicted in Figure 4C show that both the IC_50_ values and the δmP decreased along with the decreased concentration of mAb. In all the mAb dilutions assessed, the 1/2500 for the mAb 5B2 was utilized due to it producing the lowest IC_50_/δmP.

#### 3.3.4. The Study of Reaction Kinetic of the Competition

**Figure 5:**
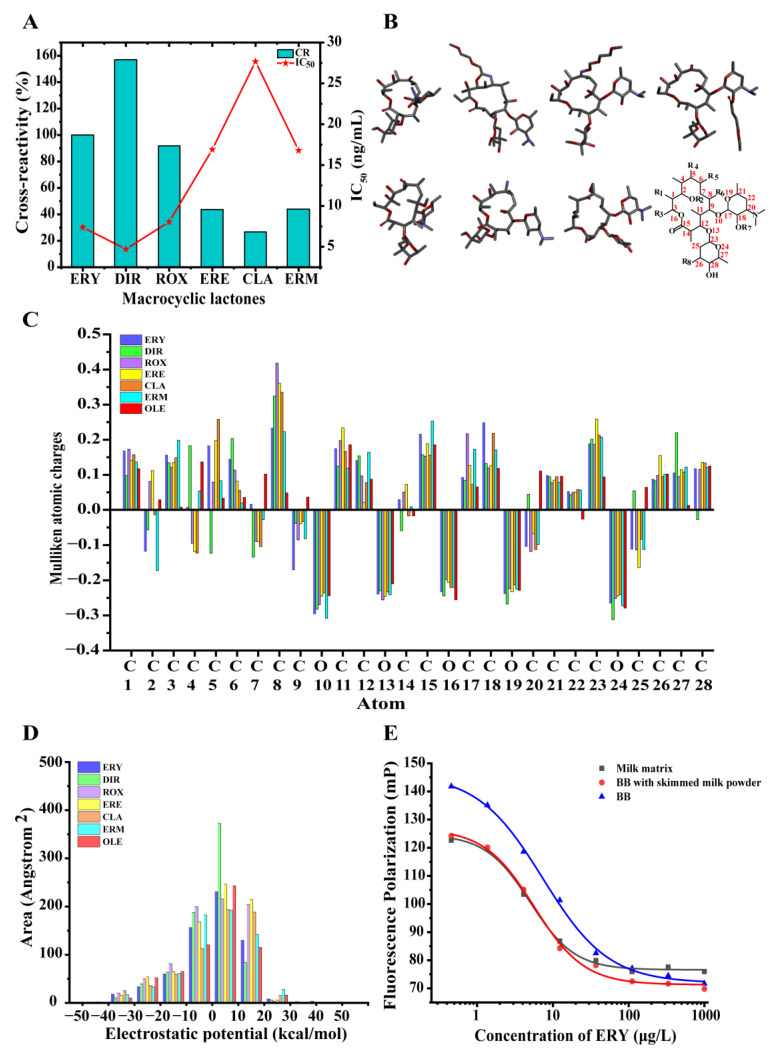
(**A**) The CR of macrocyclic lactones in FPIA; (**B**) lowest energy conformation of ERY, DIR, ROX, ERE, CLA, ERM, OLE, and the numbers of the main skeleton C atoms of seven analytes; (**C**) calculated partial atomic Mulliken charges of the numbers shown in (**B**); (**D**) superficial area in ESP range on the van der Waals surface of ERY and analogs; (**E**) calibration curves of the FPIA for ERY.

The FP signal varied over time until the competitive reaction equilibrium was achieved among the standard ERY, mAb 5B2, and ERM-FITC. A kinetic study of FPIA was performed from 5 to 30 min. As presented in Figure 4D, it was found that the IC_50_/δmP were comparative stabile from 0.11 to 0.15 as incubation times varied from 5 to 30 min. Therefore, it can be concluded that the equilibrium of the reaction was reached after 5–10 min of incubation. Thus, 5 min was employed as the optimal reaction time. Under the optimal conditions, the calibration curve of the FPIA for ERY was established with an IC_50_ of 7.39 ng/mL in buffer, as depicted in Figure 5.

#### 3.3.5. Specificity of the FPIA

To evaluate the specificity of the constructed FPIA (expressed by its CR), 12 macrolides (DIR, ROX, ERE, CLA, ERM, KIT, JOS, OLE, IVM, AVE, TYL, and SPI) and two pleuromutilins (VAL and TAM) were tested. As shown in Figure 5A and Table S1, the FPIA method showed an obvious CR with the macrolide antibiotics with 14-member lactone rings, i.e., ERY (100%), DIR (156.9%), ROX (91.8%), ERE (43.7%), CLA (26.7%), and ERM (44.0%), except for OLE (<0.1%). Other macrolide antibiotics containing 16-membered lactone rings (KIT, JOS, IVM, AVE, TYL, and SPI) or the pleuromutilins (VAL and TAM) were not recognized by the antibody (CR < 0.1%). The antibody–antigen recognition was primarily determined by molecular shape and electrical properties [29]. In this study, the number of lactone ring atoms and substituent groups inevitably changed the conformation and electron distribution of the macrolide antibiotic, meaning they exert a great influence on antibody recognition. The existence of a 14-membered lactone ring in the macrolide can produced high antibody affinities. However, we speculated that the lack of one methyl group on L-cladinose of OLE (red circle, Table S1) resulted in no antibody binding even though OLE does possess a 14-membered lactone ring.

To further understand the displayed CRs, we conducted a computational chemistry analysis to further compare the structural difference of the seven analytes with a 14-membered lactone ring. These analytes were acquired under the lowest conformations, and only the backbone of these analogues was shown without hydrogen atoms. The observed molecular shape among the seven analytes were significantly different and may affect antibody–antigen recognition, and therefore affecting uniform recognition (Figure 5B). Furthermore, the other significant factor of antibody recognition is considered to be the electronic contribution of small molecules. We provided the numbers of the main skeleton C atoms of the seven analytes (Figure 5B) and analyzed the electron distribution of C26, where OLE lacks one methyl group. As shown in Figure 5C, the charges on C26 of the seven analytes were similar. This result demonstrates that the lack of the methyl group did not greatly change the atomic charges of OLE. Therefore, it is not the main factor affecting antigen and antibody binding. As displayed in Figure 5D, the area distributions of different electrostatic potential (ESP) intervals on the van der Waals surface were measured. The figure demonstrates that the surface areas in the different ESP ranges were mainly between −10 and 20 kcal/mol. The data demonstrated no discernable difference from each other. In conclusion, the results of the conformations and electronic properties indicated that the recognition of 14-membered lactone ring except OLE by the mAb may be mainly due to shape matching.

### 3.4. Analysis of Milk Samples

FPIA is a rapid, high-throughput, and robust method for high-throughput detection of samples. Milk is a very complex solution consisting of many components, such as fats, proteins, and sugars, which may affect the quantification of the target by impacting its specific recognition by the antibody [33,34]. Reducing the impact of the matrix is necessary to obtain a high accuracy and precision. Traditional methods for extracting antibiotics from milk involve precipitating the proteins with organic solvents. Ethyl acetate was considered for extracting ERY [13]; however, the main chemical properties of ERY is unstable in acidic media [35,36], and has a low recovery of less than 60%. Various solvents were applied to the extraction of macrolide compounds such as saturated ammonium sulfate and trichloroacetic acid, but the recoveries were as low as that with ethyl acetate. Acetonitrile can be employed for the extraction since it provides an effective protein precipitant. However, the high concentration of organic solvent may reduce the affinity of the mAb, resulting in inaccurate detection. Thus, the extract was frequently required to be evaporate to near dryness under a gentle stream of nitrogen which was very time-consuming. To improve the extraction yield and minimize matrix effects, 10% acetonitrile-BB with the same good extraction effect was applied for extracting ERY from milk. Compared to the BB calibration curve, the milk matrix reduced the signal value (δmP from 74.2 to 47.0), but it had the effect of improving the sensitivity of the FPIA (IC_50_ from 7.39 ng/mL to 4.95 ng/mL, Table S2). By comparing the milk matrix calibration curve with the BB calibration curve with added 0.4% skimmed milk powder (mass fraction), the working ranges of 2.02–12.14 ng/mL and 1.95–16.39 ng/mL, respectively, were almost overlapped indicating that the aforementioned sample pretreatment was feasible. The detailed results can be seen in Figure 5E and Table S2.

A calibration curve with 0.4% skimmed milk powder (mass fraction) was performed to analyzing the ERY-spiked milk for the evaluation of sensitivity, accuracy, and precision. The LOD of ERY in milk was calculated to be 14.08 μg/L with a detection range of 25.06–235.76 μg/L, which was sensitive enough to achieve the detection requirements of MRL for ERY in milk set by the EU and China. The blank milk was spiked with ERY at 50, 100, and 150 μg/L, and the ERY measurement was carried out with the developed FPIA after pretreatment. The results shown in Table 2 suggested that the mean recoveries of the milk samples were 96.08–107.77%, with a CV between 3.41% and 10.97%. These results indicated that the established FPIA supplied an acceptable performance in terms of specificity, accuracy, and precision for detecting ERY residues in milk. The LOD of the ELISA with 0.3 μg/L was lower than that of the developed FPIA with 14.08 μg/L using the same mAb 5B2 in milk [28]; however, the homogeneous FPIA demands a much shorter time of about 5 min for the detection of ERY in milk, which is urgently required for rapid screening methods. Table S3 shows the LOD and assay time of the developed FPIA compared with typical instrumental analytical methods and immunoassays [1,12,13,28,36,37,38], demonstrating that the newly established FPIA had the shortest detecting time for ERY under a satisfactory LOD, which confirmed the suitability of this assay for the rapid, accurate, and precise determination of ERY in real samples.

## 4. Conclusions

In summary, we have developed a homologous FPIA with great detecting performances, low cost, and time-savings for the analysis of ERY in milk. The more sensitive FPIA was acquired under an optimal antibody–tracer pair, physicochemical conditions, and reaction time. As a homogeneous method, the assay exhibited time efficiency within 5 min while avoiding coating and washing, which was a unique advantage for the rapid screening of ERY. The reliability and robustness of the developed FPIA, including the LOD, detectable range, specificity, accuracy, and precision, confirmed the suitability of this assay as a tool for the rapid, simple, sensitive, and high-throughput screening of ERY in milk.

## Figures and Tables

**Table 1:** Analytical parameters of each antibody–tracer pair in buffer.

Tracers	mAbs	δmP (mP)	Antibody Dilution	IC_50_ (ng/mL)
ERY-CMO-4′-AMF	mAb 5B2	145	1/1500	80.3
	mAb 6C2	164	1/2500	107.4
	mAb 6D9	183	1/2000	92.0
ERM-FITC	mAb 5B2	210	1/1000	24.0
	mAb 6C2	109	1/1200	154.3
	mAb 6D9	225	1/1700	34.7
ERM-SRSC	mAb 5B2	97	1/600	62.1
	mAb 6C2	90	1/1600	390.0
	mAb 6D9	76	1/500	159.5
ERM-AF647	mAb 5B2	156	1/3000	13.0
	mAb 6C2	177	1/10,000	85.0
	mAb 6D9	142	1/3000	65.9

**Table 2:** Recoveries and CVs for ERY in spiked milk simples by FPIA.

Sample	LOD (μg/L)	Spiked Level (μg/L)	Recovery (%)	CVs (%)
milk	14.08	50	107.77	6.15
		100	97.33	3.41
		150	96.08	10.97

## Data Availability

Data is contained within the article or Supplementary Materials.

## Supplementary Materials

The following supporting information can be downloaded at: https://www.mdpi.com/article/10.3390/foods12081581/s1, Figure S1: The TLC purification of tracers; Figure S2: Mass spectra of ERY-CMO-4′-AMF(A), ERM-FITC (B), and ERM-SRSC (C); Figure S3: Binding curves for three antibodies with the ERY-CMO-4′-AMF (A), ERM-FITC (B), ERM-AF647 (C), and ERM- SRSC (D); Figure S4: Standard curves for three antibodies with ERY-CMO-4′-AMF (A), ERM-FITC (B), ERM-SRSC (C), and ERM-AF647 (D); Table S1: The IC_50_ values and CRs of FPIA; Table S2: The parameters of standard curves in BB, BB with skimmed milk powder, and milk matrix; Table S3: Summary of typically reported immunoassays for the determination of ERY. References [1,12,13,28,36,37,38] are cited in the Supplementary Materials.
