# Supplementary material for: A Robust Homogeneous Fluorescence Polarization Immunoassay for Rapid Determination of Erythromycin in Milk
Source: Foods. 2023 Apr 7;12(8):1581. doi: 10.3390/foods12081581 (PMC10138142; doi:10.3390/foods12081581)
Supplement: Supplementary file 1 [file foods-12-01581-s001.zip › foods-2296153-supplementary.pdf]

Supporting information for

A Robust Homogeneous Fluorescence Polarization Immunoassay for Rapid Determination of Erythromycin in Milk

Changfei Duan[1], Huiyan Zhang[1], Yingjie Zhang[1], Qiang Li[1], Peipei Li[1], Ghulam Mujtaba Mari[2], Sergei A. Eremin[3], Jianzhong Shen[1], Zhanhui Wang[1*]

[1] National Key Laboratory of Veterinary Public Health Security, Beijing Key Laboratory of Detection Technology for Animal-Derived Food, College of Veterinary Medicine, China Agricultural University, 100193 Beijing, People's Republic of China

[2] Department of Veterinary Pharmacology and Toxicology, Faculty of Bio-Sciences, Shaheed Benazir Bhutto University of Veterinary and Animal Sciences, Sakrand, 67210. Pakistan

[3] Department of Chemistry, M.V. Lomonosov Moscow State University, 119991 Moscow, Russia

**Corresponding author: Zhanhui Wang*

Tel: +86-10-6273 4565

Fax: +86-10-6273 1032

E-mail:wangzhanhui@cau.edu.cn

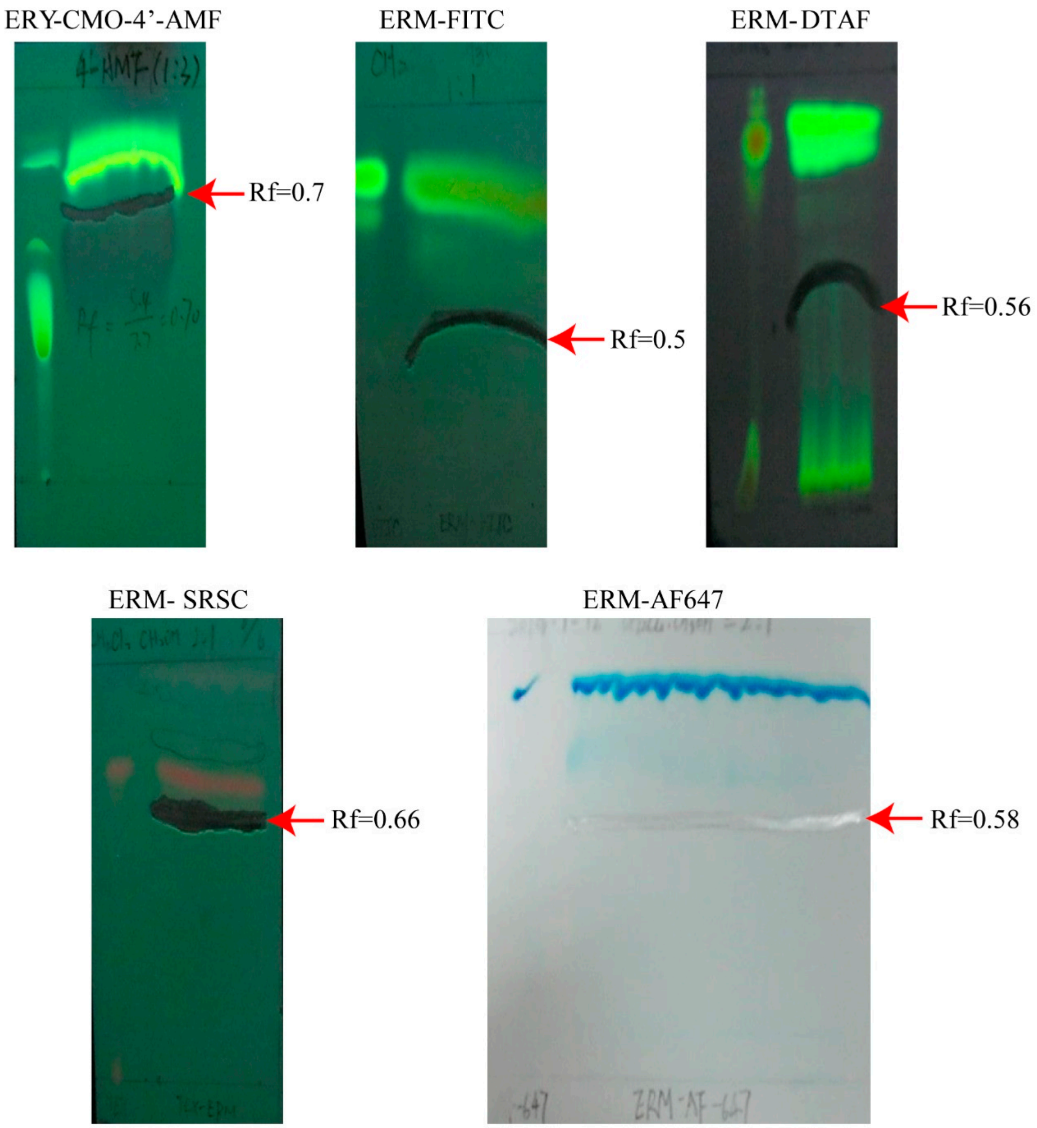

Figure S1 The TLC purification of tracers.

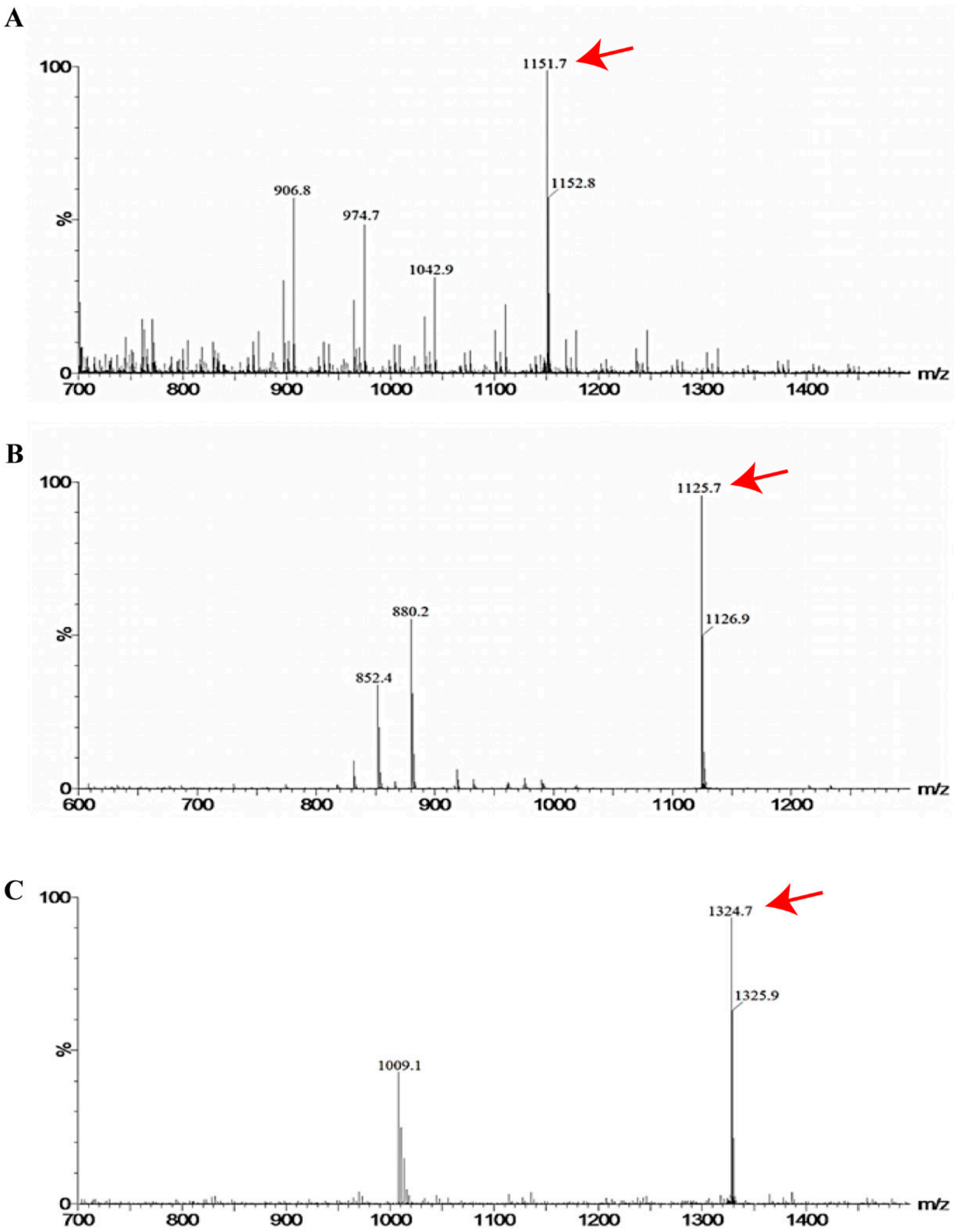

Figure S2 Mass spectra of the ERY-CMO-4'-AMF(A), ERM-FITC (B) and ERM-SRSC (C).

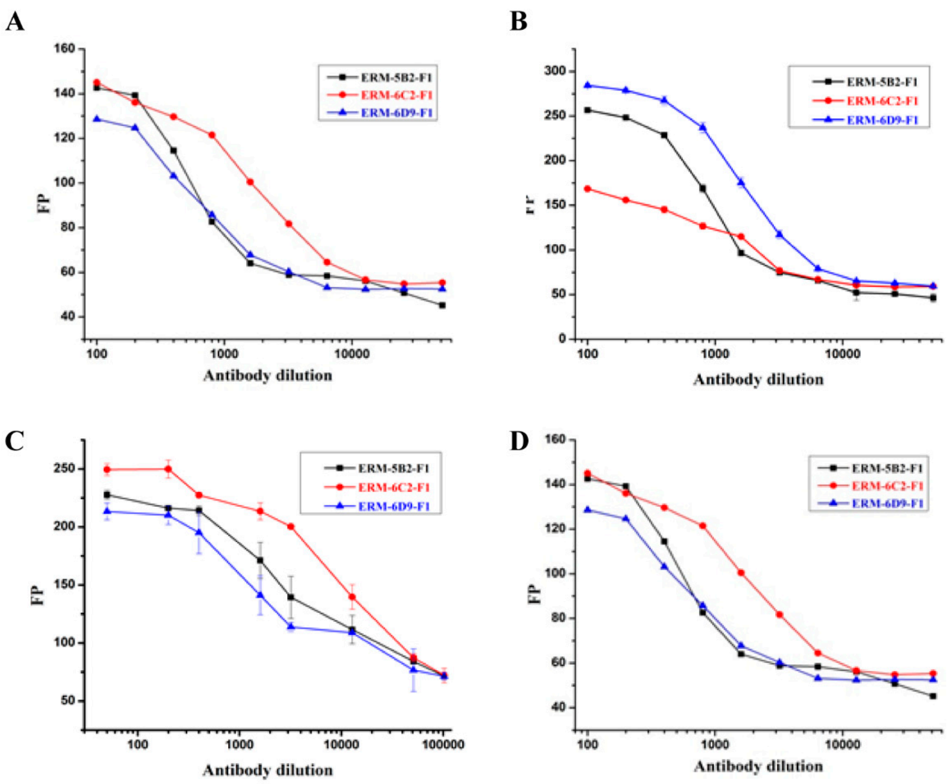

Figure S3 Binding curves for three antibodies with the ERY-CMO-4'-AMF (A), ERM-FITC (B), ERM-AF647 (C) and ERM- SRSC (D), respectively.

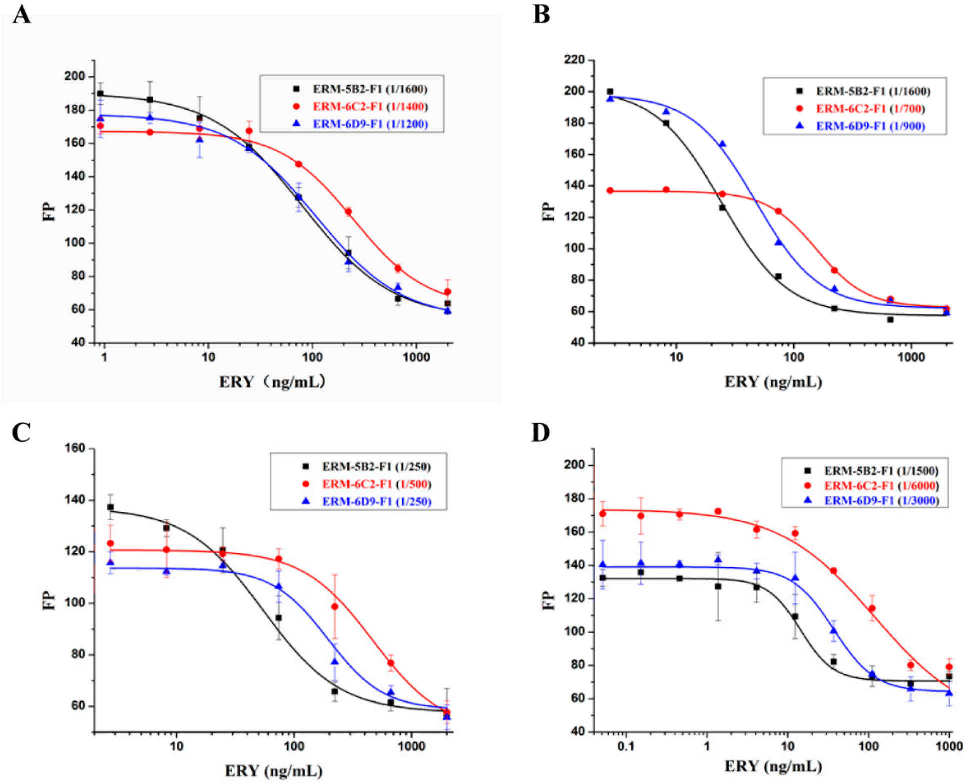

Figure S4 Standard curves for three antibodies with ERY-CMO-4'-AMF (A), ERM-FITC (B), ERM-SRSC (C) and ERM-AF647 (D).

Table S1. The IC_{50} values and CRs of FPIA.

Type	Name	Structures	IC_{50} （ng/mL）	CRs （%）
14-membered macrolides	ERY	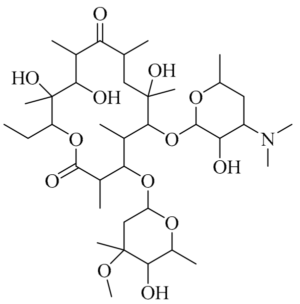	7.39	100.0
	DIR	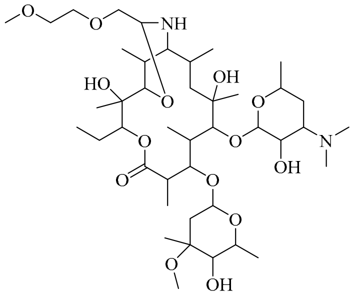	4.71	156.9
	ROX	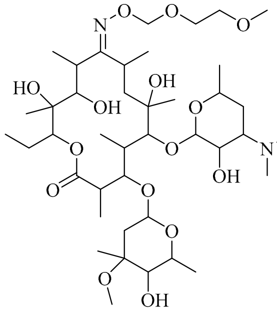	8.05	91.8
	ERE	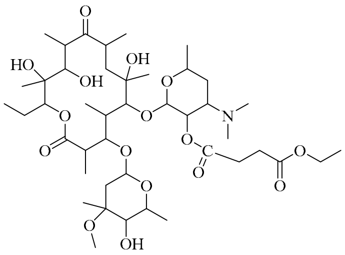	16.91	43.7
	CLA	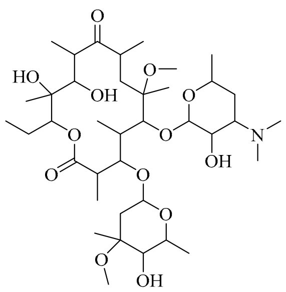	27.67	26.7

	ERM	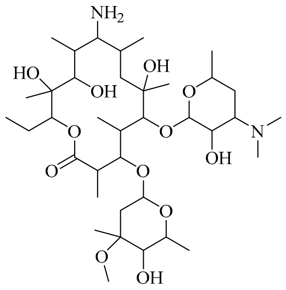	16.79	44.0
	OLE	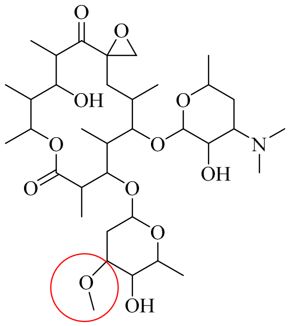	—	< 0.1
16-membered macrolides	KIT	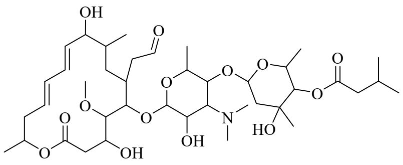	—	< 0.1
	JOS	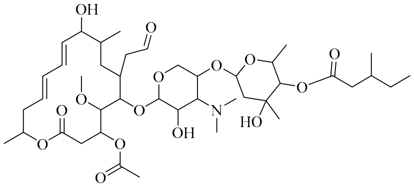	—	< 0.1
	IVM	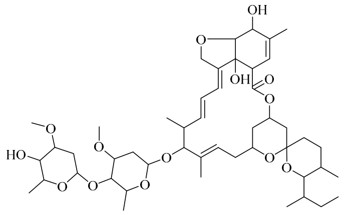	—	< 0.1
	AVE	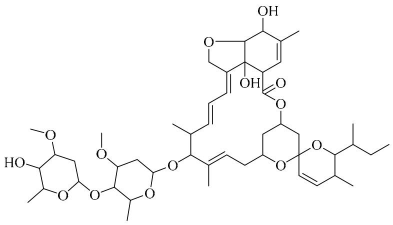	—	< 0.1
	TYL	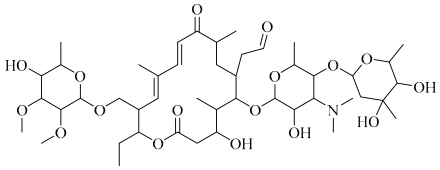	—	< 0.1

	SPI	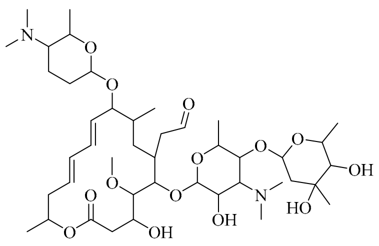	—	< 0.1
pleuromutilin	VAL	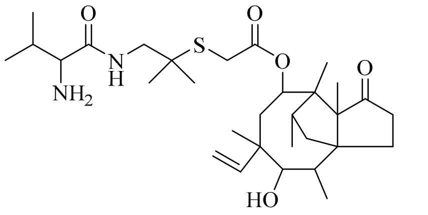	—	< 0.1
	TAM tiamulin	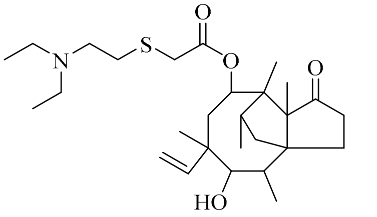	—	< 0.1

Table S2. The parameters of standard curves in BB, BB with skimmed milk powder, and milk matrix, respectively.

Standard curve	δmP (mP)	IC_{50} （ng/mL)	IC_{10} (ng/mL)	IC_{20}-IC_{80} （ng/mL)	R^2
Milk matrix	47.0	4.95	1.2	2.02-12.14	0.9971
BB with skimmed milk powder	55.8	5.66	1.05	1.95-16.39	0.9931
BB	74.2	7.39	0.83	1.85-29.47	0.9969

Table S3. Summary of typically reported immunoassays for the determination of ERY.

Methods	Samples	LOD/LOQ (μg/L or μg/kg)	Assay Time (from added samples)	Reference
ciELISA	milk	0.3	2h 30 min	[28]
ciELISA	milk	0.03	2h 40 min	[36]
IGCA	milk	5	10 min	[37]
MISPE-HPLC-ELSD	milk	10	15 min	[13]
HPLC	milk	25	15 min	[1]
MISPE-LC-MS/MS	muscle	1.0	15 min	[12]
LC–MS/MS	muscle	5	20 min	[38]
FPIA	milk	14.08	**5 min**	**This study**

ciELISA: competitive indirect enzyme-linked immunosorbent assay

IGCA: immunogold chromatographic assay

MSPE-HPLC-ELSD: molecularly imprinted polymers solid phase extraction combining with High Performance Liquid Chromatography-Evaporation Light Scattering Detection

HPLC: High Performance Liquid Chromatography

MISPE-LC-MS/MS: Molecularly imprinted polymer solid phase extraction combining with LC–MS/MS

LC–MS/MS: liquid chromatography–tandem mass spectrometry
